# The Relationship between the Physical Activity Environment, Nature Relatedness, Anxiety, and the Psychological Well-being Benefits of Regular Exercisers

**DOI:** 10.3389/fpsyg.2017.01058

**Published:** 2017-06-26

**Authors:** Emma Lawton, Eric Brymer, Peter Clough, Andrew Denovan

**Affiliations:** ^1^Department of Sport, Manchester Metropolitan UniversityManchester, United Kingdom; ^2^Institute of Sport, Physical Activity and Leisure, Leeds Beckett UniversityLeeds, United Kingdom; ^3^Department of Psychology, University of HuddersfieldHuddersfield, United Kingdom; ^4^Department of Psychology, Manchester Metropolitan UniversityManchester, United Kingdom

**Keywords:** anxiety, natural environment, nature relatedness, physical activity, well-being

## Abstract

Research from a variety of scientific fields suggests that physical activity in nature and feelings of connection to nature enhance psychological health and well-being. This study investigated the psychological health and well-being impact of the physical activity environment for those already undertaking the recommended weekly amount of physical activity. This topic is important for the design of health and well-being environments and interventions involving physical activity. Participants (*N* = 262) aged 18–71 years (*M* = 34.5, *SD* = 13.1) who met the UK physical activity guidelines completed the Nature Relatedness Scale, the trait section of the State Trait Inventory for Cognitive and Somatic Anxiety and the Psychological Well-Being Scale. Analysis via Multivariate ANOVA indicated that participants who engaged in outdoor physical activity reported significantly lower somatic anxiety levels and higher Nature Relatedness experience (NRexp). Significant results were not evident for wellbeing. Hierarchical regressions revealed that the psychological well-being facet of autonomy, NRexp, and outdoor physical activity predicted lower somatic anxiety, whereas indoor physical activity predicted higher somatic anxiety. Results indicate that somatic anxiety is lower for outdoor physical activity participation, and that outdoor activity, in conjunction with autonomy and NRexp, predicts lower anxiety levels. The findings extend previous work by demonstrating the impact of the physical activity environment on anxiety levels, as well as the contribution of outdoor physical activity and well-being facets to the previously established Nature Relatedness-anxiety relationship.

## Introduction

In recent years, there has been a growing body of evidence suggesting that time spent in the presence of nature improves psychological health and well-being (Pretty, 2004; Bowler et al., 2010; Shanahan et al., 2016). For example, Chang and Chen (2005) found that window views of nature and indoor plants were related with low anxiety related behaviors. Tension and anxiety increased when window views of nature and indoor plants were removed. Weinstein et al. (2009) found that exposure to natural environments enhanced caring behaviors and psychological well-being. Mitchell (2013) found an association between regular use of natural environments for leisure activities and a lower risk of mental health issues. However, the association between natural environments and health outcomes might be more complex than initially understood (Ward Thompson and Aspinall, 2011; Mitchell, 2013). Much of the previous research measured short-term benefits of physical activity interventions in natural environments and little is known about the previous physical activity habits of participants. This study aimed to investigate the psychological health and well-being impact of the physical activity environment for those already undertaking the recommended weekly amount of physical activity.

### The role of physical activity in nature

The benefits of physical activity in nature, such as walking in forests, gardening, and outdoor activities have been well-documented (Pretty et al., 2007; Page, 2008; Ryan et al., 2010; Pasanen et al., 2014; Passmore and Howell, 2014). Physical activity in nature has been associated with enhanced mood (Hartig et al., 2003), improvements in attentional capacity (Berman et al., 2008), and cognitive capacity (Berman et al., 2012). For example, Ryan et al. (2010) determined that the presence of nature while walking mediated vitality. Pretty et al. (2007) found that physical activities such as horse riding, walking, cycling, fishing, and conservation activities in nature led to significant improvements in self-esteem and total mood disturbance. Hartig et al. (2003) found that when compared to walking in urban areas walking in the presence of nature enhanced positive emotional and cognitive outcomes. Passmore and Howell (2014) investigated the effect of a 2-week physical activity in nature intervention on eudemonic (meaning and self-realization) and hedonic (pleasure attainment and pain avoidance) well-being. They found that both eudemonic well-being and hedonic tone were enhanced after the 2-week intervention. Mayer et al. (2009) undertook a study comparing a 15-min walk in a natural setting with a similar walk in an urban setting. Emotional well-being was enhanced by exposure to actual nature, compared to urban settings.

While many studies that have examined the psychological health and well-being benefits of physical activity in nature have focused on the enhancement of positive outcomes, some studies have considered the benefits of green exercise for the reduction of psychological discomfort such as stress and anxiety. Anxiety has been categorized as consisting of two distinct facets. State anxiety describes the temporary anxiety experienced when in direct relationship to immediate perceptions of threat. Trait anxiety is a relatively durable characteristic that underlies the intensity of and tendency for state anxiety responses (Spielberger and Reheiser, 2009). High trait anxiety can determine state anxiety occurrences. Studies that have examined the relationship between anxiety and green exercise have focused on the benefits of short bouts of green exercise on state anxiety and found that the greenness of the environment while exercising was more likely to be related to reductions in state anxiety than exercise on its own (Mackay and Neill, 2010). Regular exercise has also been associated with lower trait anxiety (Paluska and Schwenk, 2000) and as a result researchers have proposed that participation in regular green exercise is likely to be related to low levels of trait anxiety (Pretty et al., 2007; Martyn and Brymer, 2016). Studies have also found that nature can help manage and reduce the effects of stress, which if chronic might lead to anxiety (Vyas et al., 2004). For example, studies have shown that even one bout of physical activity in a natural environment can have long-term benefits for stress (Park et al., 2010; Korpela et al., 2014; Takayama et al., 2014). These studies suggest that nature can reduce the potential long term, harmful effects of chronic stress and interfere with the potential for stress to lead to anxiety.

However, despite the number of studies showing improvements in psychological health and well-being through nature-based physical activities the exact role and impact of the natural environment in this process is still rather unclear (Karmanov and Hamel, 2008; Kjellgren and Buhrkall, 2010; Keniger et al., 2013; Brymer et al., 2014; Yeh et al., 2016b). For example, Martens et al. (2011) investigated the influence of wild compared to tended forest environments and found that well-being, positive affect and negative affect showed stronger positive changes in the tended forest condition. This would suggest that benefits obtained from physical activity in nature have a ceiling effect where more accessible natural environments are more appropriate for enhancing positive affect and well-being than wild environments. Loureiro and Veloso (2014) found that for regular exercisers a combination of outdoor and indoor exercise was significantly associated with positive affect and well-being, suggesting that, for regular exercisers, the greatest influence on psychological wellbeing might be physical activity. Kerr et al. (2006) compared laboratory and natural settings on the emotions of recreational and competitive runners and found that both conditions facilitated equivalent beneficial effects. Kerr et al. concluded that the importance of the exercise environment for regular exercisers might be overstated.

Research has demonstrated that even one bout of physical activity at moderate levels has a considerable benefit for psychological health and well-being (Ekkekakis et al., 2000). As most studies assessing the benefits of green environments for exercise have not ascertained if participants were currently meeting physical activity recommendations it is feasible that the findings reflect take up of physical activity and nature provides a pleasant environment whereby participants are able to regulate physical activity at moderate to pleasant levels. Nature, like music, might just be one of many possible ways to distract attention toward pleasant stimuli (Yeh et al., 2016a). And, like music, individual differences in the recipient might impact on the efficacy of nature as an intervention. Indeed, one size may well not fit all; for example, it might be that the benefits found in studies to date are influenced by the participants' psychological make up. One possibly important factor is their experiences of connectedness to nature (Martyn and Brymer, 2016).

### The role of connection to nature

A strand of research exploring the health benefits of nature is beginning to show that individual differences in affective, cognitive, and experiential connections with the natural world influence the benefits found (Zelenski and Nisbet, 2012). For example, Zhang et al. (2014) found a positive relationship between individual experiences of connectedness to nature and life satisfaction and high self-esteem. Research conducted by Cervinka et al. (2011) found a relationship between psychological well-being and feelings of connectedness to nature. They also found a significant positive correlation between connectedness to nature and vitality. Martyn and Brymer (2016) found that connection to nature (Nature Relatedness) was significantly correlated with lower levels of overall anxiety, state cognitive anxiety, and trait cognitive anxiety. Furthermore, Martyn and Brymer (2016) demonstrated that Nature Relatedness (specifically Nature Relatedness experience) significantly predicted lower anxiety levels, thereby establishing an important link between Nature Relatedness and anxiety. However, physical activity was not examined as a contributing factor here. The results from these studies suggest that an individual's engagement with and connection to nature might be an important factor when studying the effects of physical activity in natural environments. A study undertaken in Portugal with regular exercisers found that well-being benefits for those who combine outdoor and indoor exercise were predicated on feelings of connectedness to nature (Loureiro and Veloso, 2014). As previously outlined, outdoor physical activity may produce positive psychological responses, however an individual's experience of connection to the natural world might be a key factor in this relationship. No study has, however, simultaneously assessed the effects of connection to nature and outdoor physical activity in relation to psychological health outcomes.

The current study aimed to investigate the role of the physical activity environment in relation to connection to nature, positive psychological health, and anxiety for regular exercisers. Additionally, this study aimed to extend the work of Martyn and Brymer (2016) by assessing the contribution of the exercise environment to the established Nature Relatedness-anxiety relationship. There were two hypotheses: (1) Individuals who regularly undertake nature-based physical activity will have higher levels of well-being and lower levels of trait anxiety than those who regularly undertake physical activity in indoor environments (2) Nature Relatedness (NR) and outdoor physical activity will positively impact on well-being and/or anxiety levels.

## Method

### Participants

Participants (*N* = 262) consisted of 102 men and 160 women, aged 18–71 years (*M* = 34.5, *SD* = 13.1). The inclusion criteria for the study were that participants were adults (19–64 years) and that they met the physical activity guidelines recommended by the UK Chief Medical Officer (150 min of physical activity per week). Recruitment involved sending a standard email asking for volunteers to participate in a research study looking into the psychological experiences gained from being physically active in different environments through social media (e.g., facebook) and professional networks (e.g., linkedin, the student services of the university of the first author) in the U.K. and internationally. Participants were recruited based on their regular exercise habits rather than their relationship to the natural world. Participation was not linked to compensation. This study was carried out in accordance with the recommendations of the Manchester Metropolitan University ethics procedure, approved by the Manchester Metropolitan University ethics committee with written informed consent from all participants. All participants gave written informed consent in accordance with the declaration of Helsinki. All participants acknowledged that they were over 18 and provided their consent before completing the online survey.

### Materials

An online survey was developed using a recognized free web-based survey development tool. The survey included a section on demographics, questions about physical activity choices, the environment in which this activity takes place as well as validated measures of psychological health and well-being. The physical activity questions asked participants to state their main physical activity and the environment questions asked (1) an open question that asked participants where they undertook their chosen physical activity, and (2) whether the environment was central to their physical activity of choice. Three environment options were provided and participants were asked to choose one of the three options: indoors, outdoors where nature is incidental to the experience (incidental meaning that the natural world was not essential to the experience), or outdoors where nature is central to the experience (central meaning where the natural environment is essential and important to the experience).

Three existing quantitative scales were utilized, the Nature Relatedness Scale (NRS) (Nisbet et al., 2009), the trait section of the State Trait Inventory for Cognitive and Somatic Anxiety (STICSA) (Ree et al., 2008) and the Psychological Well-Being Scale (PWB) (Ryff, 1989). The NRS is a 21-item scale that measures individuals' affective, cognitive, and physical relationship with the natural world. The NRS consists of three subscales: an 8-item subscale “self” which measures personal connection to nature, a 7-item subscale “perspective” which measures external worldviews of nature, and a 6-item subscale “experience” which measures physical familiarity with nature (Nisbet et al., 2009). Each subscale uses a 5-point Likert scale ranging from 1 (*disagree strongly*) to 5 (*agree strongly*). The total score is calculated by averaging all 21 items (after reverse scoring appropriate items), whereby higher scores indicate a stronger connection with nature. Nisbet and Zelenski (2013) found NR to be correlated with behavior, environmental scales, and frequency of time in nature, thus supporting the reliability and validity of the NRS. Cronbach's alpha indicated satisfactory internal reliability for the NR total scale, α = 0.87. For the subscales, alpha was satisfactory for nature relatedness experience (NRexp; α = 0.70) and nature relatedness self (NRself; α = 0.87). Alpha was lower than 0.7 for nature relatedness perspective (NRpers; α = 0.60), but this can still be considered marginal reliability (Hair et al., 2006).

The trait half of the STICSA comprises of a 21-item scale with each item rated on a 4-point Likert scale, ranging from 1 (*not at all*) to 4 (*very much so*). The STICSA produces a score from 42 to 168 whereby higher scores indicate higher anxiety (Ree et al., 2008). The scale assesses an individuals' general mood state and predicts the situations in which different individuals will display heightened state anxiety (Ree et al., 2008). Within the trait anxiety scale, anxiety is then further categorized as two subscales comprising of 11 somatic items and 10 cognitive items. Trait somatic anxiety refers to physical symptoms that are generally experienced, such as feeling dizzy, tense, and suffering from a fast heartbeat. Trait cognitive anxiety refers to general feelings of worry, concern and intrusion of unpleasant thoughts. Cronbach's alpha reported satisfactory internal consistency for the total STICSA (α = 0.90), for somatic trait anxiety (SOManx; α = 0.81), and for cognitive trait anxiety (COGanx; α = 0.89).

The Psychological Well-Being Scale developed by Ryff (1989) is a 42-item scale with each item rated on a 6-point Likert scale, ranging from 1 (*strongly disagree*) to 6 (*strongly agree*). The questionnaire is designed to assess how people perceive aspects of their own functioning, e.g., do they feel that what they do in life is meaningful (Abbot et al., 2006). The scale consists of six separate dimensions: autonomy, environmental mastery, personal growth, positive relations with others, purpose in life, and self-acceptance (Ryff, 1995). Each dimension represents a distinct facet of psychological wellbeing. Cronbach's alpha reported satisfactory internal reliability for autonomy (α = 0.73), personal growth (α = 0.70), positive relations (α = 0.75), purpose in life (α = 0.70), and self-acceptance (α = 0.86). Environmental mastery was lower than 0.7 (α = 0.60), but this can still be considered marginal reliability (Hair et al., 2006).

### Statistical analysis

Specific analytic strategies were employed to address the study hypotheses. Firstly, descriptive statistics were computed to examine means, standard deviation, and assumptions among the study variables. Next, the aim was to assess whether individuals who regularly undertake nature-based physical activity have higher levels of well-being, lower anxiety, and higher nature relatedness than those who regularly undertake physical activity in indoor environments (hypothesis one). Three separate MANOVAs were employed; the first focused on anxiety outcomes (COGanx and SOManx), the second assessed wellbeing outcomes (autonomy, environmental mastery, personal growth, positive relations, purpose in life, self-acceptance), and the third assessed nature relatedness (NRself, NRpers, and NRexp) in relation to types of physical activity environment (indoor, outdoor incidental, and outdoor central). Prior to analysis, five participants were removed from the dataset due to extreme scores (i.e., z scores less than −3.25 and/or greater than 3.25; Tabachnick and Fidell, 2001) leaving a final sample of 257. Initial analysis using the Kolmogorov-Smirnov test revealed that the data were not normally distributed. Given this test can be overly sensitive with relatively large samples (Peat and Barton, 2005), skewness and kurtosis were also examined with some values falling beyond the recommended interval of −2 to +2, indicating skewed data (Byrne, 2010). To compensate for this feature of the data, bootstrapping was employed which involved resampling and replacing the original dataset 1,000 times prior to rerunning the MANOVAs. Bootstrapping is often used in research situations with non-normal data and has been shown to be effective for generating accurate confidence intervals for means (see Wang, 2001). The bias-corrected method was used to adjust parameter estimates, standard errors, and effect sizes.

To examine whether Nature Relatedness (NR) and outdoor physical activity positively impact on anxiety and/or well-being (hypothesis two), a series of hierarchical regressions were employed following examination of zero-order correlations. The hierarchical format was constructed in a way that extends the previous work of Martyn and Brymer (2016); specifically, by including NR factors of NRself, NRpers, and NRexp in the first stage of the regression as predictors of anxiety (COGanx and SOManx). In the second stage, physical activity environment was included (using dummy coding), and in the third stage facets of well-being were included (autonomy, environmental mastery, personal growth, positive relations, purpose in life, self-acceptance). Incorporating physical activity environment and well-being facets in latter stages of the analysis enabled an assessment of whether these variables meaningfully contributed to the established NR-anxiety relationship by Martyn and Brymer (2016). Well-being facets were examined in relation to NR and anxiety at the correlation stage. To account for non-normality when computing the correlations and hierarchical regressions, bootstrapping was employed with 1,000 resamples.

## Results

Table 1 presents descriptive statistics of the subscales for NR, STICSA, and well-being, as well as summary statistics of physical activities the participants engaged in. Of the final sample, the indoor group comprised 92 participants (38 men, 54 women) with an age range of 17–70 (*M* = 33.86, *SD* = 12.86). The outdoor incidental group consisted of 71 participants (32 men, 39 women) with an age range of 18–63 (*M* = 36.85, *SD* = 13.44). The outdoor central group comprised 94 participants (30 men, 64 women) with an age range of 18–71 (*M* = 33.04, *SD* = 12.63). Higher NR was evident for outdoors central physical activity compared with indoors and outdoors incidental physical activity. Compared with a sample of 305 university students (Martyn and Brymer, 2016), participants scored generally lower across environment groups for NRself (outdoors central *M* = 3.34, outdoors incidental *M* = 3.16, indoors *M* = 3.17, compared with *M* = 3.74). For NRpers, sample scores were comparable with Martyn and Brymer (2016) for both outdoors groups (outdoors central *M* = 3.92, outdoors incidental *M* = 3.83, compared with *M* = 3.98), but were lower for indoors (*M* = 3.73). Similarly, for NRexp, sample scores were comparable for both outdoors groups (outdoors central *M* = 3.67, outdoors incidental *M* = 3.56, compared with *M* = 3.70), and were lower for indoors (*M* = 3.37).

**Table 1:** Descriptive statistics for scales, physical activity type, and physical activity duration for each environment group (*N* = 257).

**Variable**	**Outdoors central (*****n*** = **94)**			**Outdoors incidental (*****n*** = **71)**			**Indoors (*****n*** = **92)**		
	***M***	***SD***	**95% BCa CI**	***M***	***SD***	**95% BCa CI**	***M***	***SD***	**95% BCa CI**
NR Total	3.62	0.62	3.49, 3.74	3.50	0.61	3.35, 3.65	3.42	0.61	3.31, 3.53
NRpers	3.92	0.58	3.80, 4.03	3.83	0.62	3.68, 3.98	3.73	0.61	3.62, 3.84
NRself	3.34	0.84	3.16, 3.50	3.16	0.92	2.95, 3.40	3.17	0.83	3.02, 3.34
NRexp	3.67	0.72	3.49, 3.82	3.56	0.62	3.41, 3.70	3.37	0.81	3.22, 3.53
Total (S)TICSA	36.19	10.44	34.02, 38.13	33.08	9.03	30.99, 35.51	37.47	10.05	35.34, 39.58
COGanx	19.12	7.10	17.61, 20.52	17.12	5.94	15.79, 18.57	19.30	6.96	17.96, 20.63
SOManx	17.06	4.72	16.11, 17.93	15.95	3.97	15.05, 16.97	18.17	4.61	17.24, 19.10
Autonomy	29.57	5.70	28.38, 30.64	30.36	5.55	29.06, 31.64	29.44	6.15	28.12, 30.67
Environmental mastery	29.05	4.92	28.02, 30.08	30.43	4.44	29.39, 31.45	29.52	4.22	28.67. 30.32
Personal growth	34.31	4.78	33.39, 35.25	33.66	5.54	32.32, 34.95	33.80	4.51	32.87, 34.72
Positive relations	32.62	5.72	31.49, 33.83	33.38	5.83	31.94, 34.75	32.66	5.35	31.59, 33.79
Purpose in life	30.97	5.17	29.90, 32.08	31.40	5.60	30.11, 32.70	31.82	5.23	30.71, 32.95
Self-acceptance	29.72	6.95	28.33, 31.10	30.38	6.12	28.92, 31.85	29.50	7.26	27.88, 31.12
Main physical activity/sport			Running (35% of group)			Running (44% of group)			Gym exercise (32% of group)
Exercise duration of main activity (mins per week)			120+ minutes (61% of group)			120+ minutes (65% of group)			120+ minutes (62% of group)
Exercise duration of main activity (years)			1–2 years (20% of group)			1–2 years (31% of group)			1–2 years (28.3% of group)

*M, Mean; SD, Standard Deviation. 95% BCa CI, Bias-corrected and Accelerated confidence interval based on 1,000 bootstrapped samples; NR Total, Nature Relatedness Total; NRpers, Nature Relatedness perspective; NRself, Nature Relatedness self; NRexp, Nature Relatedness experience; STICSA, State Trait Inventory for Cognitive and Somatic Anxiety; COGAnx, trait cognitive anxiety; SOManx, trait somatic anxiety*.

Higher anxiety levels were evident generally for indoors physical activity, whereas lower anxiety scores were evident for outdoors incidental. Compared with normative data of the STICSA (*N* = 278; Grös et al., 2007), participants scored similarly on COGanx for all environment groups (outdoors central *M* = 19.12, outdoors incidental *M* = 17.12, indoors *M* = 19.30, compared with *M* = 17.20). Participants scored lower on SOManx in the outdoors incidental group compared with normative data (*M* = 15.95, compared with *M* = 17.10), but this was not the case for outdoors central (*M* = 17.06) or indoors (*M* = 18.17).

Wellbeing scores did not appear to be markedly different across the three physical activity environments, and were similar to normative data (Widdowson et al., 2016). In relation to physical activity, the main activity reported for the indoor group (62%) was gym exercise (including weight training, powerlifting, circuit training, gym classes). For the outdoor incidental and outdoor central groups, the main physical activity was running, with a higher frequency reported for the outdoor incidental group (44% compared with 35%). The majority of participants across the activity environments reported engaging in their main activity for more than 120 min per week. Responses for the duration of the main activity ranged from a minimum of 5 months (weight training; indoor group) to 35 years (horse riding; outdoor central). Interestingly, shorter durations were typically reported for indoor main activities compared with outdoor main activities.

### Hypothesis one: nature relatedness, anxiety, and well-being as a function of different physical activity environments

Application of MANOVA with bootstrapping revealed a significant main effect of activity on anxiety, Wilks' λ = 0.95, *F*_(4, 506)_ = 2.84, *p* = 0.024, η_*p*_^2^ = 0.02 (small effect size). Levene's test was non-significant for COGanx and SOManx (*p* = 0.150 and *p* = 0.127 respectively), as was Box's M (*p* = 0.085), indicating the variance-covariance matrices are homogenous (Stevens, 2002). Tests of between-subjects effects revealed significant differences for activity and SOManx, *F*_(2, 254)_ = 4.92, *p* = 0.008, η_*p*_^2^ = 0.04 (small effect size). Bootstrap estimates, using indoor physical activity as a reference category, indicated that participants engaging in outdoor incidental physical activity had significantly lower SOManx than participants engaging in indoor physical activity, BCa 95% CI of −3.42 to −0.99, *p* = 0.002. No significant difference was observed for outdoor central physical activity compared with indoor physical activity.

MANOVA with bootstrapping reported no significant main effect of activity on wellbeing facets, Wilks' λ = 0.95, *F*_(12, 498)_ = 1.08, *p* = 0.373, η_*p*_^2^ = 0.02. In addition, no significant between-subjects effects were observed for activity and well-being facets. Levene's test was non-significant for all well-being variables, as was Box's M (*p* = 0.051). Bootstrapping estimates are not reported given the absence of a main effect. MANOVA with bootstrapping revealed a significant main effect of activity on NR, Wilks' λ = 0.94, *F*_(6, 504)_ = 2.38, *p* = 0.028, η_*p*_^2^ = 0.03 (small effect size). Levene's test was non-significant for all NR variables, as was Box's M (*p* = 0.116). Tests of between-subjects effects revealed significant differences for activity and NRexp, *F*_(2, 254)_ = 3.82, *p* = 0.023, η_*p*_^2^ = 0.03 (small effect size). Bootstrap estimates, with indoor physical activity as a reference category, indicated that participants engaging in outdoor central physical activity had significantly higher NRexp than participants engaging in indoor physical activity, BCa 95% CI of 0.07 to 0.50, *p* = 0.016. No significant difference was observed for outdoor incidental physical activity compared with indoor physical activity for NRexp.

### Hypothesis two: nature relatedness, type of physical activity, and well-being as predictors of anxiety

Correlations between NR total, NR subscales, STICSA total, STICSA subscales, and well-being facets were examined using the bivariate bootstrap technique to compensate for non-normality (Rasmussen, 1987). The results for each correlation are shown in Table 2. Significant negative correlations were found between NR total, NRexp and NRself with anxiety outcomes. In addition, all well-being facets significantly negatively correlated with anxiety outcomes. Only the autonomy wellbeing subscale was significantly positively correlated with NR, specifically NRexp, *r*_(255)_ = 0.13, BCa 95% CI of 0.01 to 0.25, *p* = 0.044.

**Table 2:** Correlation coefficients for scales and subscales with accompanying 95% BCa CI (*N* = 257).

**Variable**	**1**	**2**	**3**	**4**	**5**	**6**	**7**	**8**	**9**	**10**	**11**	**12**	**13**
1. NR total		0.69^**^ [0.61,0.76]	0.82^**^ [0.77,0.87]	0.89^**^ [0.86,0.91]	−0.19^**^ [−0.32,−0.06]	−0.16^**^ [−0.29,−0.03]	−0.18^**^ [−0.29,−0.07]	0.09 [−0.03,0.19]	0.09 [−0.02,0.19]	0.02 [−0.11,0.13]	0.01 [−0.13,0.13]	0.06 [−0.07,0.18]	0.07 [−0.05,0.19]
2. NRpers			31^**^ [0.18,0.44]	0.48^**^ [0.38,0.57]	−0.08 [−0.20,0.04]	−0.08 [−0.21,0.06]	−0.06 [−0.16,0.06]	−0.03 [−0.15,0.09]	−0.03 [−0.12,0.07]	−0.03 [−0.15,0.09]	−0.02 [−0.12,0.10]	−0.04 [−0.14,0.09]	0.01 [−0.11,0.12]
3. NRself				0.71^**^ [0.63,0.78]	−0.22^**^ [−0.35,−0.09]	−0.18^**^ [−0.30.−0.04]	−0.23^**^ [−0.35,−0.11]	0.13^*^ [0.01,0.25]	0.11 [−0.01,0.23]	0.05 [−0.08,0.18]	0.01 [−0.11,0.16]	0.09 [−0.05,0.21]	0.09 [−0.03,0.22]
4. NRexp					0.17^**^ [−0.29,−0.05]	−0.15^*^ [−0.26,−0.02]	−0.17^**^ [−0.27,−0.05]	0.10 [−0.01,0.21]	0.10 [−0.01,0.22]	0.02 [−0.01,0.13]	0.01 [−0.11,0.13]	0.09 [−0.05,0.21]	0.06 [−0.05,0.19]
5. Total (S)TICSA						0.89^**^ [0.85,0.92]	0.83^**^ [0.77,0.87]	−0.48^**^ [−0.57,−0.38]	−0.49^**^ [−0.59,−0.37]	−0.34^**^ [−0.45,−0.22]	−0.35^**^ [−0.48,−0.23]	−0.40^**^ [−0.51,−0.28]	−0.63^**^ [−0.69,−0.54]
6. COGanx							0.55^**^ [0.45,0.65]	−0.48^**^ [−0.57,−0.38]	−0.47^**^ [−0.58,−0.35]	−0.32^**^ [−0.43,−0.20]	−0.35^**^ [−0.47,−0.23]	−0.39^**^ [−0.51,−0.26]	−0.66^**^ [−0.73,−0.59]
7. SOManx								−0.35^**^ [−0.45,−0.25]	−0.38^**^ [−0.49,−0.26]	−0.27^**^ [−0.39,−0.14]	−0.26^**^ [−0.38,−0.15]	−0.30^**^ [−0.43,−0.19]	−0.39^**^ [−0.49,−0.29]
8. Autonomy									0.48^**^ [0.36,0.59]	0.46^**^ [0.35,0.56]	0.35^**^ [0.23,0.46]	0.36^**^ [0.24,0.47]	0.51^**^ [0.42,0.60]
9. Environmental mastery										0.47^**^ [0.34,0.59]	0.55^**^ [0.44,0.65]	0.64^**^ [0.55,0.72]	0.65^**^ [0.56,0.74]
10. Personal growth											0.54^**^ [0.42,0.64]	0.65^**^ [0.56,0.73]	0.62^**^ [0.52,0.70]
11. Positive relations												0.57^**^ [0.47,0.65]	0.61^**^ [0.52,0.68]
12. Purpose in life													0.67^**^ [0.58,0.74]
13. Self-acceptance													

*NR Total, Nature relatedness total; NRexp, Nature relatedness experience; NRself, Nature relatedness self; NRpers, Nature relatedness perspective; STICSA, State Trait Inventory for Cognitive and Somatic Anxiety; COGAnx, trait cognitive anxiety; SOManx, trait somatic anxiety*.

* *p < 0.05*,

** *p < 0.001*.

Assumptions of collinearity, homoscedasticity, independence of errors, and absence of outliers were assessed prior to the hierarchical regression analyses, with no notable issues. The first hierarchical regression with bootstrapping examined NR (Stage 1), indoor physical activity (Stage 2), and well-being facets (Stage 3) as predictors of SOManx. Stage 1 reported an *R*^2^ of 0.05, accounting for 5% of the variance in anxiety. The model was, however, significant, *F*_(3, 253)_ = 4.56, *p* = 0.004. Stage 2 significantly improved on Stage 1 by including indoor physical activity (*F*_*change*_ = 5.05, *p* = 0.026) and accounted for 7% of the variance in anxiety. Including well-being facets at Stage 3 significantly improved the predictive power of the regression (*F*_*change*_ = 9.57, *p* < 0.001), with the model accounting for 24.6% of the variance in anxiety. Bootstrap estimates revealed that at Stage 3, indoor physical activity significantly predicted higher SOManx (*B* = 1.24, BCa 95% CI of 0.17 to 2.33, *p* = 0.024), and the autonomy facet of well-being significantly predicted lower SOManx (*B* = –0.12, BCa 95% CI of –0.24 to –0.02, *p* = 0.026). Interestingly, prior to Stage 2, NRexp significantly predicted lower SOManx (*B* = –1.35, BCa 95% CI of –2.39 to –0.21, *p* = 0.012).

The second hierarchical regression with bootstrapping examined NR (Stage 1), outdoor central physical activity (Stage 2), and wellbeing facets (Stage 3) as predictors of SOManx. As with the first hierarchical regression, Stage 1 accounted for 5% of the variance in SOManx (*R*^2^ = 0.05). The inclusion of outdoor central physical activity at Stage 2 did not significantly contribute to the regression model (*F*_*change*_ = 0.06, *p* = 0.80). Inclusion of wellbeing at Stage 3 significantly improved the regression overall (*F*_*change*_ = 9.50, *p* < 0.001) and the final model explained 23% (*R*^2^ = 0.23) of SOManx. Bootstrap estimates revealed that at Stage 3, NRexp significantly predicted lower SOManx (*B* = −1.06, BCa 95% CI of −1.94 to −0.91, *p* = 0.036), and the autonomy facet of well-being significantly predicted lower SOManx (*B* = −0.13, BCa 95% CI of −0.23 to −0.03, *p* = 0.024).

The third hierarchical regression with bootstrapping examined NR (Stage 1), outdoor incidental physical activity (Stage 2), and well-being facets (Stage 3) as predictors of SOManx. Stage 1 accounted for 5% of the variance in SOManx (*R*^2^ = 0.05). Inclusion of outdoor incidental physical activity at Stage 2 significantly contributed to the regression model (*F*_*change*_ = 6.95, *p* = 0.009), with the model accounting for 8% of variance in SOManx (*R*^2^ = 0.08). Inclusion of well-being at Stage 3 significantly improved the regression (*F*_*change*_ = 9.12, *p* < 0.001) and the final model explained 25% (*R*^2^ = 0.25) of SOManx. Bootstrap estimates at Stage 3 revealed that outdoor incidental significantly predicted lower SOManx (*B* = −1.27, BCa 95% CI of −2.42 to −0.14, *p* = 0.024). NRexp significantly predicted lower SOManx (*B* = −0.99, BCa 95% CI of −1.93 to −0.10, *p* = 0.045), and the autonomy facet of well-being significantly predicted lower SOManx (*B* = −0.12, BCa 95% CI of −0.24 to −0.02, *p* = 0.025) (see Table 3).

**Table 3:** Hierarchical regression predicting somatic anxiety from NR subscales, outdoor incidental physical activity (dummy coded), and well-being subscales (*N* = 257).

**Variable**	**Step 1**			**Step 2**			**Step 3**		
	***B***	***SE (B)***	**BCa 95% CI**	***B***	***SE (B)***	**BCa 95% CI**	***B***	***SE (B)***	**BCa 95% CI**
NRpers	0.13	0.47	−0.89, 1.05	0.18	0.47	−0.78, 1.02	−0.11	0.44	−1.01, 0.79
NRexp	−1.35^*^	0.50	−2.27, −0.28	−1.23^*^	0.49	−2.15, −0.21	−0.99^*^	0.48	−1.93, 0.10
NRself	−0.09	0.41	−0.85, 0.66	−0.22	0.40	−0.98, 0.50	−0.03	0.42	−0.82, 0.67
Outdoor incidental physical activity				−1.63^*^	0.58	−2.77, −0.42	−1.27^*^	0.54	−2.42, −0.14
Autonomy							−0.12^*^	0.05	−0.24, −0.02
Environmental mastery							−0.14	0.09	−0.34, 0.06
Personal growth							−0.01	0.07	−0.15, 0.12
Positive relations							0.01	0.07	−0.13, 0.12
Purpose in life							−0.01	0.08	−0.19, 0.16
Self-acceptance							−0.13	0.07	−0.26, 0.01
*R^2^*	0.05			0.08			0.25		
*F*	4.56^*^			5.24^**^			7.98^**^		
*Fchange*				6.95^*^			9.12^**^		

*NRexp, Nature relatedness experience; NRself, Nature relatedness self; NRpers, Nature relatedness perspective; BCa CI, Bias-corrected and Accelerated confidence interval based on 1,000 bootstrapped samples*.

* *p < 0.05*,

** *p < 0.001*.

The hierarchical regression analyses examining NR, types of physical activity, and well-being in relation to COGanx did not document any significant effects at Stage 1 or Stage 2 for NR and types of physical activity. Overall, the MANOVA outcomes indicate firstly that participants who engaged in outdoor physical activity (particularly incidental) had significantly lower SOManx than participants engaging in indoor physical activity. Additionally, participants engaging in outdoor central physical activity had significantly higher NRexp than participants engaging in indoor physical activity. The hierarchical regressions indicate that autonomy, NRexp, and outdoor physical activity (particularly incidental) predicted lower SOManx, whereas indoor physical activity predicted higher SOManx. These results extend previous work in the area, by revealing the added contribution of types of physical activity and well-being facets to the previously established NR-anxiety relationship.

## Discussion

This study had two main aims; the first was to investigate the impact of the physical activity environment on well-being and trait anxiety for regular exercisers. The second aim was to investigate the relationship between nature relatedness, trait anxiety, and psychological well-being in regular exercisers. There were two hypotheses: (1) Individuals who regularly undertake nature-based physical activity will have higher levels of well-being and lower levels of trait anxiety than those who regularly undertake physical activity in indoor environments, and (2) Nature Relatedness (NR) and outdoor physical activity will positively impact on well-being and/or anxiety levels. The findings partially support the first hypothesis, indicating that individuals who regularly engaged in outdoor-based physical activity had lower levels of somatic anxiety in comparison with individuals who took part in indoor-based physical activity. However, the difference between the exercise environment where nature was central to the experience and the indoor environment was apparent but not significant. In relation to hypothesis two, the findings indicate that although the activity environment was not influential relative to wellbeing facets, and only autonomy was meaningful in relation to NR; NRexp, autonomy, and outdoor physical activity predicted lower somatic anxiety, whereas indoor physical activity predicted higher somatic anxiety.

Taken together this study provides further evidence that feeling experientially connected to nature is related to some aspects of psychological well-being and low somatic anxiety. This study also suggests that for individuals who meet UK physical activity guidelines for regular physical activity the presence of the natural world might not be a central determinant for self-reported levels of trait anxiety or psychological well-being. How an individual feels toward the natural world and exercising outdoors (even if nature is not central to the experience) seems to be more important.

In relation to hypothesis one, the results show that for regular exercisers there were no differences in overall trait anxiety levels, and cognitive anxiety levels or psychological well-being levels, across all physical activity environments. There was, however, differences in somatic anxiety levels. Although previous research has reported that physical activity in the natural world conveys significantly greater psychological benefits than indoor physical activity (e.g., Passmore and Howell, 2014), the findings in this study suggest that for individuals who undertake regular physical activity the immediate exercise environment has minimal influence on wellbeing levels, but can help to lower somatic anxiety.

Psychological wellbeing levels for all participants in this study were in line with population norms. Regular physical activity has been associated with positive psychological well-being (Penedo and Dahn, 2005). A meta-analysis conducted by Penedo and Dahn (2005) concluded that all types of exercise are beneficial for a range of physical and psychological well-being outcomes. Regular physical activity has been associated with greater levels of life satisfaction, increased quality of life outcomes and increased happiness as compared to non-exercisers (Stubbe et al., 2007). Previous research examining the wellbeing benefits of nature-based exercise has most often focused on short-term green exercise interventions with participants who may not have been regular exercisers. This study indicates that for those who regularly exercise the immediate exercise environment does not seem to be a key determinant for well-being benefits. Results from this study are similar to those found by Kerr et al. (2006) who determined that for regular runners the exercise environment was minimally related to well-being outcomes. However, the rich sensory experience afforded by the natural environment might still act as a welcome distraction or motivator for those who struggle to maintain regular exercise or for those wishing to start regular exercise.

Regular physical activity has also long been associated with lower levels of overall trait anxiety irrespective of confounding factors such as age and gender (Petruzzello et al., 1991; Scully et al., 1998; De Moor et al., 2006). On average trait anxiety levels for total trait anxiety, cognitive anxiety, and somatic anxiety were comparable to normative anxiety scores for this cohort and considerably lower than clinical populations. Average scores for somatic anxiety were significantly lower for the outdoor incidental group. This is most likely because the cohort in this study were regular exercisers. Regardless of the type of anxiety measure utilized (trait or state, self-report, or behavioral), or irrespective of the exercise regime undertaken (intensity, time, type), research has consistently reported a link between lower anxiety scores and regular participation in physical activity (Landers and Petruzzello, 1994; Anderson and Shivakumar, 2013). The anxiolytic effects of regular exercise for trait anxiety are stronger and longer lasting than many traditional therapeutic processes (Anderson and Shivakumar, 2013). This is particularly the case for trait somatic anxiety as regular exercise is said to mimic many of the physiological responses to anxiety (e.g., rapid heart rate, sweating, tense, or weak muscles and feeling hot) rendering them less potent (Anderson and Shivakumar, 2013). However, the significantly lower scores recorded for the outdoor incidental group and the lower (but not statistically significant) scores for the outdoor central group might be related to being comfortable exercising in all weathers and conditions. It is possible that exercising outdoors is associated with being more comfortable with uncomfortable somatic and sensory experiences. As with well-being, the natural environment might still facilitate motivation and act as a distractor that encourages continued participation and therefore the anxiolytic benefits of regular exercise.

Hypothesis two was partially supported in that there were negative correlations between NRtotal, NRexp, and NRself and trait anxiety, and positive correlations between NRexp and the autonomy subscale for psychological wellbeing. The subscale measuring external worldviews on nature (NRpers) was not correlated with well-being or anxiety. NRexp and autonomy were significantly related with lower anxiety levels. Another point of particular interest was that even though anxiety levels for this cohort were similar to normative, non-clinical values there were still significant negative correlations between overall trait anxiety and overall nature relatedness, and between trait cognitive anxiety, trait somatic anxiety, and NRtotal, NRexp, and NRself.

Furthermore, NRexp, autonomy, and the outdoor physical activity environment (specifically incidental) predicted lower somatic anxiety but not cognitive anxiety. These findings are in partial agreement with previous results described by Martyn and Brymer (2016) who found NRexp predicted lower anxiety (albeit cognitive), thereby establishing a link between NR and anxiety. The current study furthers understanding of this relationship by demonstrating the additional effect of the outdoor physical environment and feelings of autonomy. While regular outdoor physical activity might be beneficial for predicting lower trait anxiety, feeling part of nature, and physically comfortable in nature might have an augmenting impact. Exercising in nature all the time may not be essential if a participant feels part of nature providing a participant can exercise outdoors. The differences between the findings for NRexp, trait cognitive and trait somatic anxiety evident from this study in comparison with Martyn and Brymer (2016) might be because cognitive and somatic anxiety have different antecedents. Cognitive anxiety is more related to worry and concern whereas somatic anxiety is linked to the physical symptoms of anxiety such as butterflies in the stomach. Activities that are more related to reduced cognitive anxiety are most likely cognitive in nature such as thought reframing. Those activities most likely to facilitate low somatic anxiety are most likely oriented around using the body, such as yoga and physical activity. Somatic anxiety infers bodily discomfort such as raised heart rate, sweating and muscular changes. As noted earlier, NRexp reflects a physical familiarity with the natural world even those aspects that are not comfortable such as being out in all weathers, wilderness camping, mosquitoes, death, and decay (Nisbet et al., 2009). This would suggest that high NRexp equates to a high propensity for enduring bodily discomfort and as such the somatic response to anxiety might be less problematic.

The results concerning autonomy are interesting, and autonomy may be predictive of lower anxiety because this characteristic is synonymous with feelings of control. Indeed, research has consistently identified that feelings of control are linked with lower anxiety across various subpopulations (e.g., Fischer and Boer, 2011). In addition, recent research has shown that autonomy is predictive of improved mental health outcomes among individuals who engage in leisure-based physical activity (Denovan and Macaskill, 2016), thereby suggesting that autonomy potentially enhances the beneficial effects of physical activity on mental health outcomes, which may have occurred for the participants in this study.

Concerning the negative correlation between anxiety and NRself, NRself reflects the appreciation that as individuals we are part of nature. Nature is perceived to be spiritually rewarding. Recent studies are finding strong relationships between spiritual beliefs and lower anxiety scores (e.g., Boscaglia et al., 2005). People high in the NRself subcategory view themselves as part of nature and as spiritually connected to nature. This might explain the relationship between NRself and lower trait anxiety. Practical and clinical implications for this could be to attempt to enhance individuals' relation with nature, in order to heighten their psychological well-being levels.

For this cohort, connection to nature is related to lower anxiety and being physically comfortable exercising outdoors seems to be important. As it is often the physical relationship with nature that instigates feelings of connection to nature (Martyn and Brymer, 2016), exercising where nature is related to the experience (even incidentally) likely enhances the effects of nature connectedness (NRexp) on anxiety levels.

## Limitations

Before concluding, the authors acknowledge that the study utilized a cross-sectional design. Therefore, findings relating to the observed predictive relationships between NR and physical activity with anxiety provide only correlational evidence. Although literature and this study's findings support the direction of the observed relationships, it is important for future research to carry out longitudinal assessments to more fully examine causal relations among the variables. Furthermore, this study used self-report measures, which can be associated with issues including shared method variance and response bias. Lastly, this study used a self-selecting sample. As a result, it is possible that the study largely attracted participants who were interested in nature and were regular exercisers.

## Conclusion

Results from this study challenge the current thinking that the immediate environment in which exercise takes place is the most important factor for the psychological wellbeing outcomes produced. Instead, findings from regular exercisers in this study suggest that psychological wellbeing is similar across all exercise environments. However, both the exercise environment and the relationship an individual has with the natural environment are important with regards to anxiety levels, and feeling connected with nature and physically comfortable in nature is strongly related to autonomy and lower somatic trait anxiety. Future studies should aim to determine the direction of the NR and trait anxiety relationship. The findings reported here regarding the relationship between NR, trait anxiety and psychological wellbeing (specifically autonomy) do offer the intriguing possibility that designing interventions to enhance NR might also be useful for improving well-being and reducing anxiety.

## Author contributions

EB research design, theoretical focus, data analysis, and article development. EL Research design, data collection, data analysis, article development. PC theoretical focus, article development, analysis. AD data anlysis, theorerical perspectives, and article development.

### Conflict of interest statement

The authors declare that the research was conducted in the absence of any commercial or financial relationships that could be construed as a potential conflict of interest.

## References

[B1] Abbot R. A. Ploubidis G. B. Huppert F. A. Kuh D. Wadsworth M. E. J. Croudace T. J. (2006). Psychometric evaluation and predictive validity of Ryff's psychological well-being items in a UK cohort sample of women. Health Qual. Life Outcomes 4:76. 10.1186/1477-7525-4-76 17020614 PMC1634744

[B2] Anderson E. Shivakumar G. (2013). Effects of exercise and physical activity on anxiety. Front. Psychiatry 4:27. 10.3389/fpsyt.2013.00027 23630504 PMC3632802

[B3] Berman M. G. Jonides J. Kaplan S. (2008). The cognitive benefits of interacting with nature. Psychol. Sci. 19, 1207–1212. 10.1111/j.1467-9280.2008.02225.x 19121124

[B4] Berman M. G. Kross E. Krpan K. M. Askren M. K. Burson A. Deldin P. J.. (2012). Interacting with nature improves cognition and affect for individuals with depression. J. Affect. Disord. 140, 300–305. 10.1016/j.jad.2012.03.012 22464936 PMC3393816

[B5] Boscaglia N. Clarke D. M. Jobling T. W. Quinn M. A. (2005). The contribution of spirituality and spiritual coping to anxiety and depression in women with a recent diagnosis of gynecological cancer. Int. J. Gynecol. Cancer 15, 755–761. 10.1111/j.1525-1438.2005.00248.x 16174220

[B6] Bowler D. E. Buyung-Ali L. M. Knight T. M. Pullin A. S. (2010). A systematic review of evidence for the added benefits to health of exposure to natural environments. BMC Public Health 10:456. 10.1186/1471-2458-10-456 20684754 PMC2924288

[B7] Brymer E. Davids K. Mallabon E. (2014). Understanding the psychological health and well-being benefits of physical activity in nature: an ecological dynamics analysis. J. Ecopsychol. 6, 189–197. 10.1089/eco.2013.0110

[B8] Byrne B. M. (2010). Structural Equation Modeling with AMOS: Basic Concepts, Applications, and Programming. New York, NY: Routledge.

[B9] Cervinka R. Röderer K. Hefler E. (2011). Are nature lovers happy? On various indicators of well-being and connectedness to nature. J. Health Psychol. 17, 379–388. 10.1177/1359105311416873 21859800

[B10] Chang C. Y. Chen P. K. (2005). Human response to window views and indoor plants in the workplace. Horticult. Sci. 40, 1354–1359.

[B11] De Moor M. H. M. Beem A. L. Stubbe J. H. Boomsma D. I. De Geus E. J. C. (2006). Regular exercise, anxiety, depression and personality: a population based study. Prev. Med. 42, 273–279. 10.1016/j.ypmed.2005.12.002 16439008

[B12] Denovan A. Macaskill A. (2016). Stress, resilience and leisure coping among university students: applying the broaden-and-build theory. Leisure Stud. 1–14. 10.1080/02614367.2016.1240220

[B13] Ekkekakis P. Hall E. E. VanLanduyt L. M. Petruzzello S. J. (2000). Walking in (affective) circles: can short walks enhance affect? J. Behav. Med. 23, 245–275. 10.1023/A:1005558025163 10863677

[B14] Fischer R. Boer D. (2011). What is more important for national well-being: money or autonomy? A meta-analysis of well-being, burnout, and anxiety across 63 societies. J. Pers. Soc. Psychol. 101, 164–184. 10.1037/a0023663 21604894

[B15] Grös D. F. Antony M. M. Simms L. J. McCabe R. E. (2007). Psychometric properties of the State-Trait Inventory for Cognitive and Somatic Anxiety (STICSA): comparison to the State-Trait Anxiety Inventory (STAI). Am. Psychol. Assoc. 19, 369–381. 10.1037/1040-3590.19.4.369 18085930

[B16] Hair J. Anderson R. Tatham R. Black W. (2006). Multivariate Data Analysis, 6th Edn. Upper Saddle River, NJ: Prentice Hall.

[B17] Hartig T. Evans G. W. Jamner L. D. Davis D. S. Gärling T. (2003). Tracking restoration in natural and urban field settings. J. Environ. Psychol. 23, 109–123. 10.1016/S0272-4944(02)00109-3

[B18] Karmanov D. Hamel R. (2008). Assessing the restorative potential of contemporary urban environment(s): beyond the nature versus urban dichotomy. Landsc. Urban Plan. 86, 115–125. 10.1016/j.landurbplan.2008.01.004

[B19] Keniger L. Gaston G. Irvine K. Fuller R. (2013). What are the Benefits of Interacting with Nature? Int. J. Environ. Res. Public Health 10, 913–935. 10.3390/ijerph10030913 23466828 PMC3709294

[B20] Kerr J. Fujiyama H. Sugano A. Okamura T. Chang M. Onouha F. (2006). Psychological responses to exercising in laboratory and natural environments. Psychol. Sport Exerc. 7, 345–359. 10.1016/j.psychsport.2005.09.002

[B21] Kjellgren A. Buhrkall H. (2010). A comparison of the restorative effect of a natural environment with that of a simulated natural environment. J. Environ. Psychol. 30, 464–472. 10.1016/j.jenvp.2010.01.011

[B22] Korpela K. Borodulin K. Neuvonen M. Paronen O. Tyrväinen L. (2014). Analyzing the mediators between nature-based outdoor recreation and emotional well-being. J. Environ. Psychol. 37, 1–7. 10.1016/j.jenvp.2013.11.003

[B23] Landers D. M. Petruzzello S. J. (1994). Physical activity, fitness and anxiety, in Physical Activity, Fitness, and Health, eds Bouchard C. Shephard R. J. Stephens T. (Champaign, IL: Human Kinetics), 868–882.

[B24] Loureiro A. Veloso S. (2014). Outdoor exercise, well-being and connectedness to nature. Biling. J. Environ. Psychol. 45, 299–304. 10.13140/2.1.1219.1526

[B25] Mackay G. J. Neill J. T. (2010). The effect of ‘green exercise’ on state anxiety and the role of exercise duration, intensity, and greenness: a quasi-experimental study. Psychol. Sport Exerc. 11, 238–245. 10.1016/j.psychsport.2010.01.002

[B26] Martens D. Gutscher H. Bauer N. (2011). Walking in “wild” and “tended” urban forests: the impact on psychological well-being. J. Environ. Psychol. 31, 36–44. 10.1016/j.jenvp.2010.11.001

[B27] Martyn P. Brymer E. (2016). The relationship between nature relatedness and anxiety. J. Health Psychol. 21, 1–10. 10.1177/1359105314555169 25370570

[B28] Mayer F. S. Frantz C. M. Bruehlman-Senecal E. Dolliver K. (2009). Why is nature beneficial?: the role of connectedness to nature. Environ. Behav. 41, 607–643. 10.1177/0013916508319745

[B29] Mitchell R. (2013). Is physical activity in natural environments better for mental health than physical activity in other environments? Soc. Sci. Med. 91, 130–134. 10.1016/j.socscimed.2012.04.012 22705180

[B31] Nisbet E. K. Zelenski J. M. (2013). The NR-6: a new brief measure of nature relatedness. Front. Psychol. 4:813. 10.3389/fpsyg.2013.00813 24198806 PMC3814587

[B30] Nisbet E. K. Zelenski J. M. Murphy S. A. (2009). The nature relatedness scale: linking individuals' connection with nature to environmental concern and behavior. Environ. Behav. 41, 715–740. 10.1177/0013916508318748

[B32] Page M. (2008). Gardening as a therapeutic intervention in mental health. Nurs. Times 104, 28–30. Available online at: https://www.nursingtimes.net/gardening-as-a-therapeutic-intervention-in-mental-health/1921374.article 19051686

[B33] Paluska S. A. Schwenk T. L. (2000). Physical activity and mental health. Sports Med. 29, 167–180. 10.2165/00007256-200029030-00003 10739267

[B34] Park B. J. Tsunetsugu Y. Kasetani T. Kagawa T. Miyazaki Y. (2010). The physiological effects of Shinrin-yoku (taking in the forest atmosphere or forest bathing): evidence from field experiments in 24 forests across Japan. Environ. Health Prev. Med. 1518–26. 10.1007/s12199-009-0086-9 19568835 PMC2793346

[B35] Pasanen T. P. Tyrväinen P. Korpela K. M. (2014). The relationship between perceived health and physical activity indoors, outdoors in built environments, and outdoors in nature. Appl. Psychol. 6, 324–346. 10.1111/aphw.12031 25044598 PMC4233975

[B36] Passmore H. A. Howell A. J. (2014). Nature involvement increases hedonic and eudaimonic well-being: a two-week experimental study. Ecopsychology 6, 148–154. 10.1089/eco.2014.0023

[B37] Peat J. Barton B. (2005). Medical Statistics: A Guide to Data Analysis and Critical Appraisal. Massachusetts, NE: BMJ Books.

[B38] Penedo F. J. Dahn J. R. (2005). Exercise and well-being: a review of mental and physical health benefits associated with physical activity. Curr. Opin. Psychiatry 18, 189–193. 10.1097/00001504-200503000-00013 16639173

[B39] Petruzzello S. J. Landers D. M. Hatfield B. D. Kubitz K. A. Salazar W. (1991). A meta-analysis on the anxiety-reducing effects of acute and chronic exercise: outcomes and mechanisms. Sports Med. 11, 143–182. 10.2165/00007256-199111030-00002 1828608

[B40] Pretty J. (2004). How nature contributes to mental and physical health. Spirit. Health Int. 5, 68–78. 10.1002/shi.220

[B41] Pretty J. Peacock J. Hine R. Sellens M. South N. Griffin M. (2007). Green exercise in the UK countryside: effects on health and psychological well-being and implications for policy and planning. J. Environ. Plann. Manage. 50, 211–231. 10.1080/09640560601156466

[B42] Rasmussen J. L. (1987). Estimating the correlation coefficient: bootstrap and parametric approaches. Psychol. Bull. 101, 136–139.

[B43] Ree M. J. French D. MacLeod C. Locke V. (2008). Distinguishing cognitive and somatic dimensions of state and trait anxiety: development and validation of the state-trait inventory for cognitive and somatic anxiety (STICSA). Behav. Cogn. Psychother. 36, 313–332. 10.1017/S1352465808004232

[B44] Ryan R. M. Weinstein N. Bernstein J. Brown K. W. Mistretta L. Gagné M. (2010). Vitalizing effects of being outdoors and in nature. J. Environ. Psychol. 30, 159–168. 10.1016/j.jenvp.2009.10.009

[B45] Ryff C. D. (1989). Happiness is everything, or is it? Explorations into the meaning of psychological well-being. J. Pers. Soc. Psychol. 57, 1069–1081.

[B46] Ryff C. D. (1995). Psychological well-being in adult life. Curr. Dir. Psychol. Sci. 4, 99–104.

[B47] Scully D. Kremer J. Meade M. M. Graham R. Dudgeon K. (1998). Physical exercise and psychological well-being: a critical review. Br. J. Sports Med. 32, 111–120. 10.1136/bjsm.32.2.111 9631216 PMC1756084

[B48] Shanahan D. F. Bush R. Gaston K. J. Lin B. B. Dean J. Barber E.. (2016). Health benefits from nature experiences depend on dose. Sci. Rep. 6:28551. 10.1038/srep28551 27334040 PMC4917833

[B49] Spielberger C. D. Reheiser E. C. (2009). Assessment of emotions: anxiety, anger, depression, and curiosity. Appl. Psychol. 1, 271–302. 10.1111/j.1758-0854.2009.01017.x

[B50] Stevens J. P. (2002). Applied Multivariate Statistics for the Social Sciences, 4th Ed. Mahwah, NJ: Lawrence Erlbaum Associates.

[B51] Stubbe J. H. de Moor M. H. M. Boomsa D. I. de Geus E. J. C. (2007). The association between exercise participation and well-being: A co-twin study. Prev. Med. 44, 148–152. 10.1016/j.ypmed.2006.09.002 17059845

[B52] Tabachnick B. G. Fidell L. S (2001). Using Multivariate Statistics, 4th Edn. New York, NY: Allyn & Bacon.

[B53] Takayama N. Korpela K. Lee J. Morikawa T. Tsunetsugu Y. Park B.-J.. (2014). Emotional, restorative and vitalizing effects of forest and urban environments at four sites in Japan. Int. J. Environ. Res. Public Health 11, 7207–7230. 10.3390/ijerph110707207 25029496 PMC4113871

[B54] Vyas A. Pillai A. G. Chattarji S. (2004). Recovery after chronic stress fails to reverse amygdaloid neuronal hypertrophy and enhanced anxiety-like behavior. Neuroscience 128, 667–673. 10.1016/j.neuroscience.2004.07.013 15464275

[B55] Wang F. K. (2001). Confidence interval for the mean of non-normal data. Qual. Reliabil. Eng. Int. 17, 257–267. 10.1002/qre.400

[B56] Ward Thompson C. Aspinall P. A. (2011). Natural environments and their impact on activity, health, and quality of life. Appl. Psychol. 3, 230–260. 10.1111/j.1758-0854.2011.01053.x

[B57] Weinstein N. Przybylski A. K. Ryan R. M. (2009). Can nature make us more caring? Effects of immersion in nature on intrinsic aspirations and generosity. Pers. Soc. Psychol. Bull. 35, 1315–1329. 10.1177/0146167209341649 19657048

[B58] Widdowson M. Theuns P. Rosseau M. Rosseau R. (2016). Impact of the application of redecision methods in executive coaching workshops on psychological wellbeing: a quantitative evaluation of effectiveness. Int. J. Trans. Anal. Res. 7, 3–9. Available online at: http://usir.salford.ac.uk/40940/1/Widdowson%2C%20Theuns%2C%20Rosseau%20%26%20Rosseau%202016.pdf

[B59] Yeh H. Stone J. Churchill S. Brymer E. Davids K. (2016a). Designing physical activity environments to accrue physical and psychological effects, in Paper Presented at the 11th Conference of the International Sports Engineering Association. Delft.

[B60] Yeh H. P. Stone J. A. Churchill S. M. Wheat J. S. Brymer E. Davids K.. (2016b). Physical, psychological and emotional benefits of green physical activity: an ecological dynamics perspective. Sports Med. 46, 947–953. 10.1007/s40279-015-0374-z 26330207

[B61] Zelenski J. M. Nisbet E. K. (2012). Happiness and feeling connected: the distinct role of nature relatedness. Environ. Behav. 46, 3–23. 10.1177/0013916512451901

[B62] Zhang J. W. Howell R. T. Iyer R. (2014). Engagement with natural beauty moderates the positive relation between connectedness with nature and psychological well-being. J. Environ. Psychol. 38, 55–56. 10.1016/j.jenvp.2013.12.013

